# Structural equation modeling to shed light on the controversial role of climate on the spread of SARS-CoV-2

**DOI:** 10.1038/s41598-021-87113-1

**Published:** 2021-04-16

**Authors:** Alessia Spada, Francesco Antonio Tucci, Aldo Ummarino, Paolo Pio Ciavarella, Nicholas Calà, Vincenzo Troiano, Michele Caputo, Raffaele Ianzano, Silvia Corbo, Marco de Biase, Nicola Fascia, Chiara Forte, Giorgio Gambacorta, Gabriele Maccione, Giuseppina Prencipe, Michele Tomaiuolo, Antonio Tucci

**Affiliations:** 1 Statistics and Mathematics Area, Department of Economics, University of Foggia, Foggia, Italy; 2 Agorà Biomedical Sciences, Etromapmax Pole, Lesina (FG), Italy; 3 Department of Biomedical Sciences, Humanitas University, Via Rita Levi Montalcini, 4, 20090 Pieve Emanuele (MI), Italy; 4 Present Address: Department of Pathology, Erasmus University Medical Center, Rotterdam, The Netherlands

**Keywords:** Respiratory tract diseases, Epidemiology

## Abstract

Climate seems to influence the spread of SARS-CoV-2, but the findings of the studies performed so far are conflicting. To overcome these issues, we performed a global scale study considering 134,871 virologic-climatic-demographic data (209 countries, first 16 weeks of the pandemic). To analyze the relation among COVID-19, population density, and climate, a theoretical path diagram was hypothesized and tested using structural equation modeling (SEM), a powerful statistical technique for the evaluation of causal assumptions. The results of the analysis showed that both climate and population density significantly influence the spread of COVID-19 (p < 0.001 and p < 0.01, respectively). Overall, climate outweighs population density (path coefficients: climate vs. incidence = 0.18, climate vs. prevalence = 0.11, population density vs. incidence = 0.04, population density vs. prevalence = 0.05). Among the climatic factors, irradiation plays the most relevant role, with a factor-loading of − 0.77, followed by temperature (− 0.56), humidity (0.52), precipitation (0.44), and pressure (0.073); for all p < 0.001. In conclusion, this study demonstrates that climatic factors significantly influence the spread of SARS-CoV-2. However, demographic factors, together with other determinants, can affect the transmission, and their influence may overcome the protective effect of climate, where favourable.

## Introduction

On March 11, 2020, the respiratory disease (COVID-19) caused by the coronavirus SARS-CoV-2, after having reached a global scale, was classified by the World Health Organization as a pandemic. Still progressing, COVID-19 has completely reshaped our world from all points of view, with dramatic social, economic, and psychological consequences.

Data provided by government and health organizations show a different distribution of the epidemic across countries^1^, advocating a possible relationship between COVID-19 and climate factors. To address this issue, several studies have been carried out, but their results are mixed and conflicting, leading to deeply different conclusions^2–10^.

The reasons for such contrasting findings are several. Firstly, some studies have been designed with intrinsic limitations that do not allow for a comprehensive view of the role of climate in COVID-19 spread, whether because they considered only one or few countries, without a global perspective^3,7^, or due to short observation period chosen (2–8 weeks)^4,9,11^, or the low number of climatic variables considered (however divergent between the various investigations)^2,5^. Secondly, none of the studies published so far have considered the role played by the demographic factors. These variables are relevant to correctly interpret the apparently inconsistent results in some areas of the globe (such as India, Brazil or USA). Thirdly, procedural unintentional biases have been made in many reports^12–16^, leading to other insidious pitfalls and to the aforementioned discrepancy. One of these biases is the onset of the outbreak^12^. As the beginning of the infection did not occur simultaneously across the countries, if evaluated at the same time point, the countries whose outbreak started earlier tend to record higher prevalence of infection than the others. This requires a relative time scale, synchronizing the countries based on the beginning of the epidemic, prior to any statistical analysis.

Another pitfall is the single point of estimation^13,15^. Climatic conditions across countries vary considerably from region to region. Data collection limited to a restricted area (e.g. the capital city) is not representative of the whole country, and this may cause misleading results. Therefore, more data collection points within the same country should be taken into account. A further pitfall is the lag interval^11,14,16^. Incubation period, delayed testing from the onset of symptoms, as well as, late communication of test results, all contribute to a time shift between the infection exposure and the confirmation of diagnosis. Consequently, a lag time must be considered between the collection of climatic data for the analysis and the collection of COVID-19 data.

Finally, the interdependence of variables^17,18^. Climatic variables are commonly considered as stand-alone factors. Instead, they interact with each other. Therefore, an integrative and specific analysis is necessary for a comprehensive understanding of their effects, as their partial consideration could be misleading.

A comprehensive and detailed review of the existing literature about COVID-19 and climate is reported in the Supplementary Table S1.

Altogether, these questions may account for the discrepancy among the numerous studies published, leaving the debate about the relationship between climatic factors and COVID-19 still open.

Surely, knowing the factors influencing the epidemic and understanding the dynamics of its spread is strategic and crucial. This would allow us not only to limit the contagion, but also to better calibrate the containment policies, thus reducing the psychological, social, and economic repercussions. Starting from these considerations, we carried out an extensive and comprehensive analysis, based on as many as 134,871 data, using structural equation modeling (SEM), a statistical technique for testing the linear relationships between observed variables and latent variables, based on statistical data and causal qualitative hypothesis and developed by "LISREL" models, i.e. linear structural relationships^19–21^. Applied for the first time in the 70s in the field of social sciences with variables that could not be directly observed or measured, SEM later spread among the scientific community, thanks to both its flexibility and rigorous approach. One of the main advantages of SEM is the possibility to take into consideration several dependent variables simultaneously.

Flexibility and potential of this analysis were the key for the evaluation of climatic factors and their role in the outbreak of SARS-CoV-2, trying to overcome the limitations and the pitfalls above-mentioned. In addition, since COVID-19 seems to spread more widely in highly populated areas, we also investigated the influence of some socio-demographic variables (such as population density) over the incidence and prevalence of the disease.

## Results

### Descriptive statistics

Statistics of meteorological variables, population density, weekly incidence and prevalence of SARS-CoV-2, are summarized in Table 1. The data have been stratified by climatic zones and are relative to the first 16 weeks of infection, in the 209 countries considered in this study. From the analysis, it has emerged that there is an increasing (although apparently imperfect) trend of both incidence and prevalence from warm to cold geoclimatic areas. Low values of incidence and prevalence (both for 100,000 people) were recorded in the equatorial (μ ± σ = 4.28 ± 12.89 and μ ± σ = 11.84 ± 35.67, respectively) and arid zones (μ ± σ = 10.77 ± 34.01 and μ ± σ = 27.78 ± 95.75), areas with the hottest temperature and greater solar irradiation; while the highest values of incidence (μ ± σ = 24.68 ± 46.33) and prevalence (μ ± σ = 564.38 ± 2986.82) were observed in the warm temperate zone, the zone also recording the highest value of population density (μ ± σ = 564.38 ± 2986.82), an important factor in virus transmission through both respiratory droplets and contact routes.

**Table 1:** Summary statistics of meteorological variables, *weekly incidence* and *weekly prevalence* of SARS-CoV-2, population density, by climatic zone, in the first 16 weeks of the infection, in 209 countries.

Climatic zone	Variable	Mean	Std. Dev.	Min.	Max.
Cold temperate	Precipitation (mm/day)	2.61	2.68	0.00	18.15
	Humidity (%)	77.46	10.15	32.68	97.40
	Pressure (kPa)	96.97	5.93	78.87	102.70
	Temperature (°C)	5.85	6.11	− 15.16	25.64
	Wind (m/s)	2.80	1.62	0.28	11.59
	Solar irradiation (MJ/m^2^/day)	13.80	5.43	0.79	29.06
	Population density (n/km^2^)	58.83	62.51	3.00	284.00
	Weekly incidence (per 100,000)	23.13	34.38	0.00	205.08
	Weekly prevalence (per 100,000)	74.29	133.73	0.00	856.62
Warm temperate	Precipitation (mm/day)	2.70	3.17	0.00	21.64
	Humidity (%)	74.46	11.16	23.04	97.12
	Pressure (KPa)	97.62	4.54	76.36	102.96
	Temperature (°C)	11.72	6.41	− 11.21	32.64
	Wind (m/sec)	2.58	1.60	0.22	13.02
	Solar irradiation (MJ/m^2^/day)	15.58	5.38	1.17	29.23
	Population density (n/km^2^)	564.38	2986.82	12.00	26,337.00
	Weekly incidence (per 100,000)	24.68	46.33	0.00	342.91
	Weekly prevalence (per 100,000)	94.82	216.56	0.00	1502.94
Arid	Precipitation (mm/day)	1.58	3.76	0.00	47.56
	Humidity (%)	54.37	18.42	8.22	89.43
	Pressure (KPa)	93.93	7.99	75.22	102.37
	Temperature (°C)	19.42	8.78	− 12.36	36.95
	Wind (m/sec)	2.99	1.69	0.61	16.84
	Solar irradiation (MJ/m^2^/day)	20.15	4.47	4.09	30.69
	Population density (n/km^2^)	113.59	328.88	2.00	2239.00
	Weekly incidence (per 100,000)	10.77	34.02	0.00	425.64
	Weekly prevalence (per 100,000)	27.78	95.75	0.00	1231.56
Equatorial	Precipitation (mm/day)	5.68	7.90	0.00	61.27
	Humidity (%)	77.07	10.00	39.34	95.85
	Pressure (KPa)	98.10	4.49	83.64	101.82
	Temperature (°C)	26.18	3.03	13.30	33.25
	Wind (m/sec)	2.69	1.66	0.25	8.16
	Solar irradiation (MJ/m^2^/day)	19.54	4.32	0.77	28.37
	Population density (n/km^2^)	351.20	1082.77	8.00	8358.00
	Weekly incidence (per 100,000)	4.28	12.89	0.00	115.40
	Weekly prevalence (per 100,000)	11.84	35.67	0.00	343.64

To verify the previous findings, namely that the morbidity indices (incidence and prevalence) depend on the geoclimatic zones where the countries are geographically located, the non-parametric k-sample test for median equality has been applied. The results of the test showed statistically significant differences in incidence and prevalence among the geoclimatic zones (incidence, Chi square = 309.0387 p < 0.001; prevalence, Chi square = 317.6152 p < 0.001), confirming that the transmission of the virus is indeed influenced by the climate.

**Figure 1:**
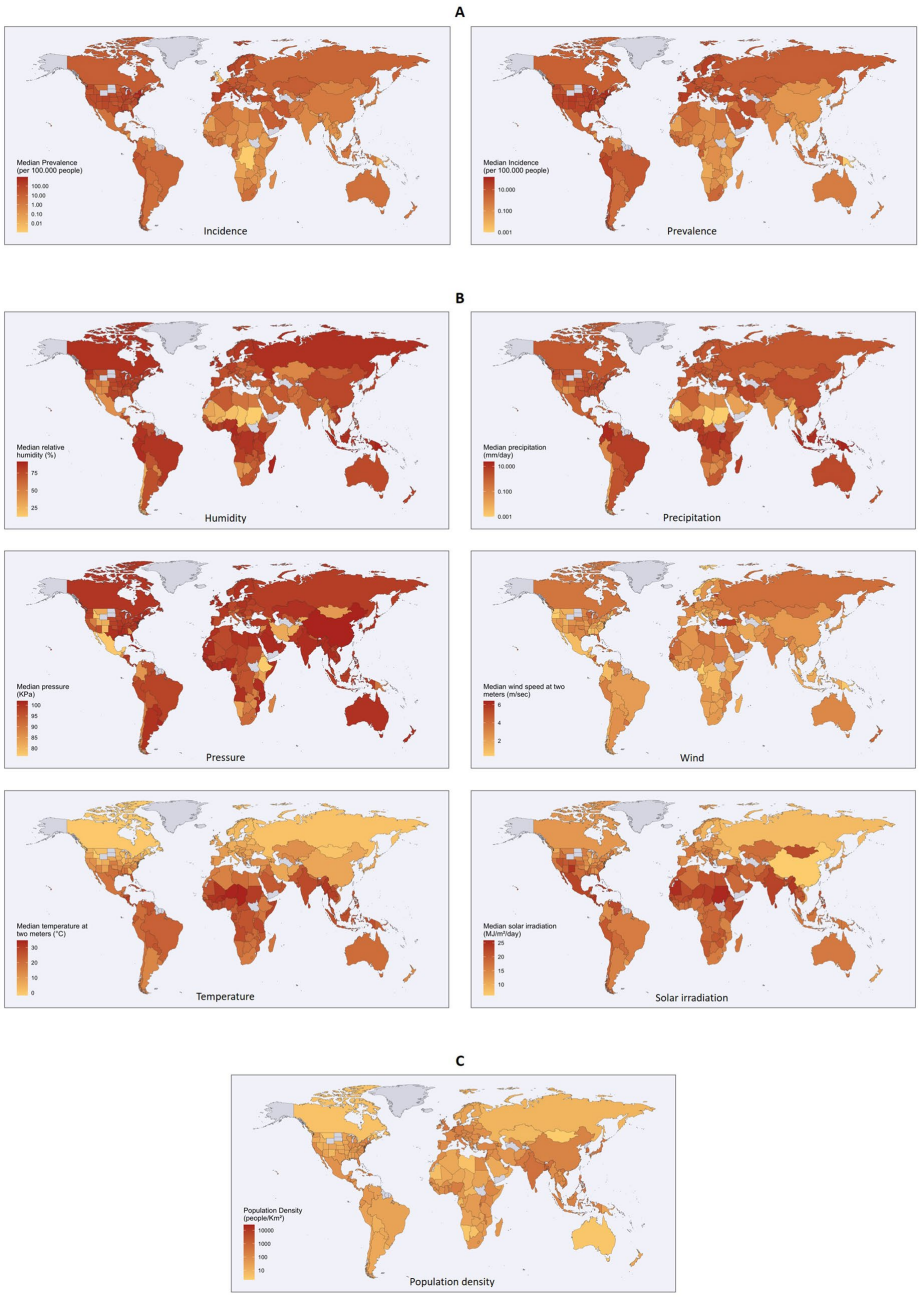
World countries maps. (**A**) Median values of incidence and prevalence of COVID-19. (**B**) Median values of the climatic variables. (**C**) Population density. In grey, countries not recorded. The maps were generated using R software (version 4.0.2—https://www.r-project.org/), ggplot2 library (version 3.3.2—https://CRAN.R-project.org/package=ggplot2) and Maps (version 3.3.0—https://CRAN.R-project.org/package=maps).

To highlight and further investigate the relationship between morbidity, climatic variables, and population density, thematic world maps have also been devised (Fig. 1). For each country, the maps illustrate the following features: (a) median incidence and prevalence values of COVID-19 in the first 16 weeks of infection, (b) median values of all climatic variables (detected with a time shift of two weeks prior to the collection of virological data) in the same period, and c) population density.

From the comparison of the maps, it resulted that, with few exceptions, no evident spatial correlation seems to exist among the investigated variables. Only solar irradiation, temperature, and humidity showed an albeit modest relationship with the morbidity indices: countries with higher solar irradiation reported lower values of incidence and prevalence, while highest incidence and prevalence values were mainly recorded in areas with lower median temperatures (e.g., United States, Europe and China) and higher humidity.

To better explore the relationship between climate and incidence/prevalence of SARS-CoV-2, the bivariate correlation between these indices and all the main meteorological variables (considered individually) was investigated by means of the Spearman's rank correlation coefficient. Then, since population density represents an important factor in the transmission of the virus, the correlation between incidence/prevalence and population density was also calculated.

The results of this analysis, in agreement with the findings of thematic world maps, demonstrated only a moderate concordance between solar irradiation and temperature (r = 0.58; p < 0.05) and a significant (albeit low) negative correlation of temperature with incidence and prevalence (r = − 0.37 and r = − 0.34, respectively) (Table 2).

**Table 2:** Spearman's rank correlation coefficient between meteorological variables, *weekly incidence* and *weekly prevalence* of SARS-CoV-2, and *population density*. *p < 0.05.

	Precipitation	Humidity	Pressure	Temperature	Wind	Solar irradiation	Population density	Incidence	Prevalence
Precipitation (mm/day)	1.00								
Humidity (%)	0.55*	1.00							
Pressure (kPa)	− 0.11	0.15*	1.00						
Temperature (°C)	− 0.02	− 0.16	0.22*	1.00					
Wind (m/s)	− 0.19	− 0.03	0.41*	0.09*	1.00				
Solar irradiation (MJ/m^2^/day)	− 0.38	− 0.50	0.05*	0.58*	0.02	1.00			
Population density (n/km^2^)	− 0.04	0.01	0.22*	0.13*	0.05*	0.11*	1.00		
Weekly incidence (per 100,000)	0.05*	0.09*	0.07*	− 0.37	− 0.01	− 0.15	− 0.01	1.00	
Weekly prevalence (per 100,000)	0.04	0.06*	0.07*	− 0.34	− 0.02	− 0.08	0.01	0.95*	1.00

However, the weak correlations detected may have been due to the heterogeneity of the climatic conditions in the numerous countries considered. In this case, a simple bivariate regression model (such as the Spearman's rank test) may be insufficient to reveal complex relationships. Therefore, a more specific analysis, in which climatic variables can be considered simultaneously and in an integrated way, is needed.

### SEM analysis

To understand the intricate interactions among geoclimatic and epidemiological variables, a more consistent and suitable mathematical model, such as the multivariate regression approach of structural equation modeling (SEM), was necessary. With this statistical model it was possible to consider the integrated effects of all the meteorological variables on COVID-19 and, at the same time, to investigate the effects of population density too.

**Figure 2:**
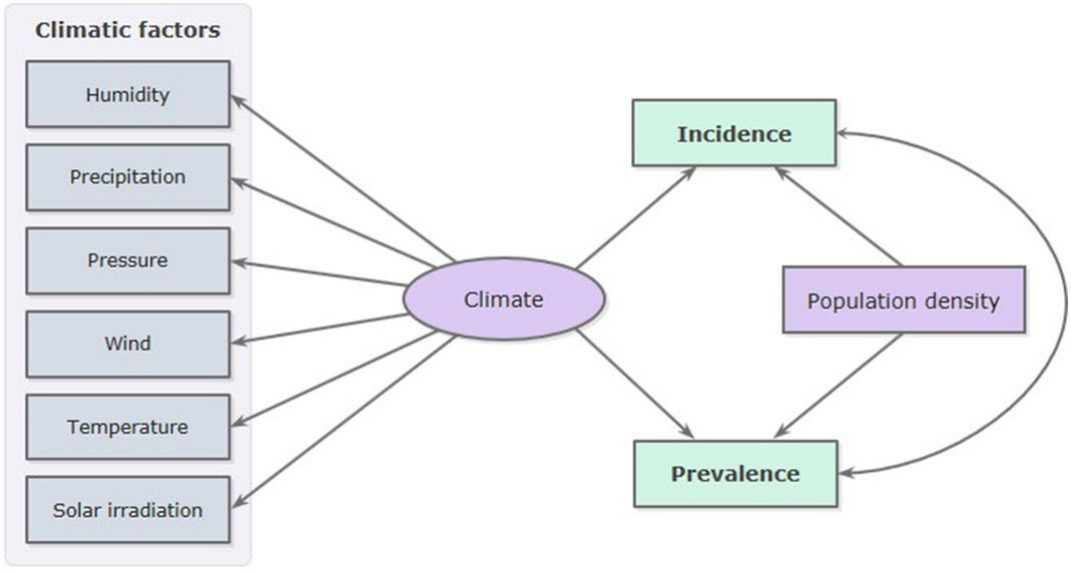
Theoretical path diagram used to analyze the effects of climate and population density on the spread of SARS-CoV-2. This diagram was generated using Office Power Point software, version 2010 (https://www.microsoft.com/it-it/microsoft-365/powerpoint).

To analyze the relations among the above-mentioned variables, the theoretical path diagram reported in Fig. 2 was supposed. In this theoretical path, meteorological factors were hypothesized to be correlated to each other and linked to a variable that cannot be directly measured (*Climate*). In addition, it was assumed that *Climate* and population density are regressors on incidence and prevalence, whose covariation is expressed by an arc. Therefore, the theoretical path was then tested by SEM.

**Figure 3:**
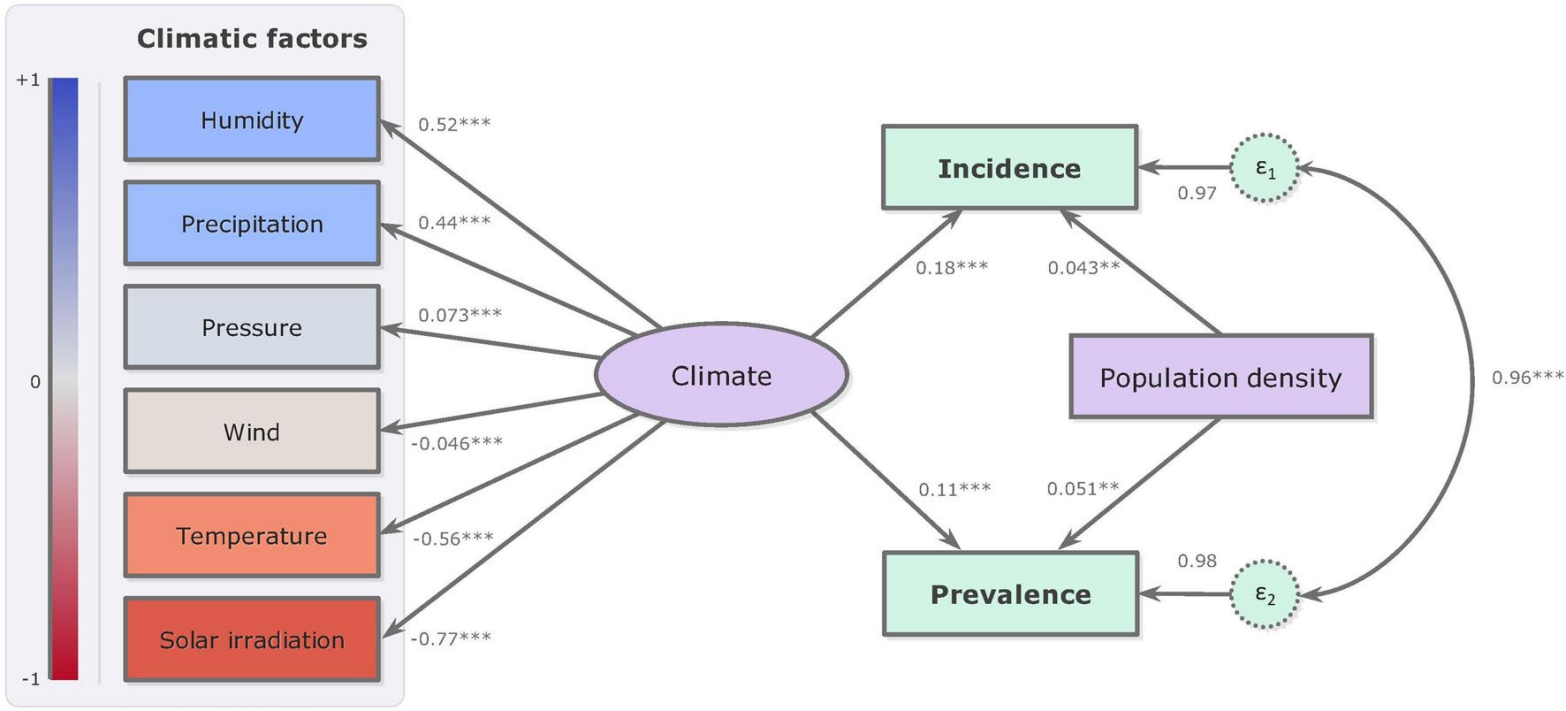
SEM path diagram for the effect of the climate and the population density on the spread of SARS-CoV-2. Observed variables are represented by boxes, latent variable by an ellipse, and residual terms by circles. The values on the straight arrows between latent and observed variables and those between latent and observed indicators represent the standardized path coefficients and the factors loading, respectively. The values on the straight arrows between residual terms and observed variables represent the residual variance. The diagram was generated using Office Power Point software for Microsoft 365 (https://www.microsoft.com/it-it/microsoft-365/powerpoint). ***p < 0.001; **p < 0.01.

The results of the analysis showed a clear causative role of climate. For paths *Climate* → *Incidence* and *Climate* → *Prevalence*, the integrated effects of meteorological factors, measured at two-week lag and expressed by a single latent variable (*Climate*), resulted to be significantly (p < 0.001) linked to incidence and prevalence by a standardized positive path coefficient (b = 0.18 and b = 0.11, respectively) (Fig. 3).

In turn, the different climatic factors were found to be correlated with *Climate* in another way. Specifically, solar irradiation and temperature were negatively correlated, with a factor loading of − 0.77 (p < 0.001) and − 0.56 (p < 0.001), respectively. While, relative humidity, precipitation and pressure showed a positive correlation, with factor loading of 0.52, 0.44, and 0.073, (for all, p < 0.001). Wind was negatively correlated (− 0.046) but without a significant p-value.

For paths *Population density* → *Incidence* and *Population density* → *Prevalence*, population density resulted to be linked to incidence and prevalence by standardized positive path coefficients (b = 0.043, p < 0.01 and b = 0.051, p < 0.01, respectively), although to a lesser extent than those exhibited by *Climate*. The variables, incidence and prevalence observed, being by their nature closely related, showed a high covariance between errors of 0.96 (p < 0.001).

An interesting outcome is given by the residual variance (var(ε_1_) = 0.97 for incidence and var(ε_2_) = 0.98 for prevalence). This parameter represents the effects of unmeasured factors influencing incidence and prevalence. It indicates that factors other than climate and population density affect SARS-CoV2 incidence and prevalence, but also random errors, errors in data entry, and systematic errors leading to biases in the measurement of values.

Finally, the goodness of fit of the model was tested, and the results of the evaluation showed an overall good fit (CD = 0.826, RMSEA = 0.088, SRMR = 0.078). This demonstrates that SEM adequately confirms the theoretical path diagram of Fig. 2, indicating the strength and direction of association between incidence and prevalence of SARS-CoV-2, climatic variables, and population density.

Overall, the results of the analysis demonstrated that (a) climate has a relevant impact on incidence and prevalence of SARS-CoV-2 (at least in the first 16 weeks), stronger than that exhibited by density of population; (b) among the climatic variables, solar irradiation proved to be the most influential factor, followed (but in the opposite direction) by relative humidity, and by temperature (in the same direction of solar irradiation).

## Discussion

COVID-19 pandemic represents a serious threat for people worldwide, with an alarming day-by-day increasing number of infections and death cases. Consequently, the scientific community worldwide is constantly seeking for new and useful knowledge aimed at contrasting the spread of SARS-CoV-2.

Climatic factors seem to play an important role in the epidemiology of the infection, although the results of the recent literature failed to give a unique and unequivocal knowledge on this subject.

In this paper, we have carried out an extensive and comprehensive analysis to determine the role of climate and some demographic factors in the spread of COVID-19. For this purpose, we used a particular and powerful statistical technique (the structural equation modeling), for the evaluation of causal assumptions, taking into consideration, as dependent variables, both weekly incidence and weekly prevalence.

The key objective of this study was to overcome the limitations of previous investigations. In line with this perspective, we have (a) conducted research on a global scale (on all countries in the world), (b) carried out observation over a long period of time (to our best knowledge, the longest among the studies published so far), (c) considered not some but all the main six climatic factors, (d) used a relative time scale, synchronizing the countries based on the beginning of the epidemic, (e) taken into account not a single point but multiple points of evaluation for climatic variables for each country, (f) considered a lag interval between the acquisition of climatic data and the collection of COVID-19 data for the analysis, (g) not considered the variables as stand-alone factors but taken into account their interdependence and used an integrative statistical investigation to evaluate their effects.

In light of this, the choice of SEM was very strategic. It allowed to test a complex model of relationships among climatic variables, population density and morbidity indices. Alternatively, several separate analyses, with less solid results in statistical terms, would have been required. The use of SEM has also the advantage of including, in the exploration model, variables (such as *Climate*) which cannot be directly observed. In addition, it solved the problem of the interdependence among the meteorological variables, allowing to address them in a comprehensive analysis.

Overall, this approach helped us to build a more solid analysis, yielding robust and reliable findings, highly consistent with the hypothesis that both climate and population density significantly influence the spread of COVID-19. Moreover, climate outweighs population density, and, in this context, solar irradiation, plays the most relevant role.

This is in line with the results of some epidemiological reports^10,11^ and with a recent experimental investigation demonstrating that UV radiation, in very small doses, is able to inactivate SARS-CoV-2^22^. Although to a lesser extent, temperature, humidity, and precipitation also resulted to significantly influence SARS-CoV2 incidence and prevalence. Instead, the role of wind was demonstrated to be small, while that of pressure was not relevant. Overall, these findings may account for the different initial distribution of the epidemic across the countries, with the cold countries being affected more quickly and more intensely than the hot ones.

Similarly, the aforementioned results provide support to the common opinion that the climate is a determining factor in the spread of the pandemic. They also provide grounds for recent clusters of infections developed during the summer in different European working places, all characterized by a lack of solar irradiation and low temperature (i.e. a sausage factory in Mantova, Italy; a slaughterhouse in Gütersloh, Germany; a slaughterhouse in Tipperary, Ireland).

However, the high rates of COVID-19 observed in the last weeks in Brazil, India, and the USA seems to contradict the conclusions drawn in the present study, as this outbreak of pandemic occurred in countries with high temperature and adequate solar irradiation. However, SEM has shown that SARS-CoV-2 incidence and prevalence are influenced not only by climate but also by population density, and that factor is very high in the three countries mentioned. Therefore, if accurately interpreted, the findings of the present study may explain this apparent paradox, as well.

A puzzling result of the SEM that needs clarifications (to avoid possible misinterpretation) is the high value of residual variance (the value on the straight arrows between residual terms and dependent variable). As stated in the Methods section the residual term (the effect of which is represented by the residual variance) includes other possible variables having effects on the dependent variable. To the untrained eye, the high value of residual variances in our study (0.97 and 0.98) seems to significantly weaken the worth of the path coefficients (having lower magnitudes: 0.04–0.18). In fact, such an approach is conceptually wrong since path coefficient and residual variance are two different entities which cannot be compared to each other. Moreover, it should not be forgotten that the residual variance includes, within itself, the effects of a lot of components (various forms of errors and other causal variables), each of which may have very little relevance. So, due to its cumulative nature, residual variance may also reach high values, without, however, affecting or weakening the worth of path coefficients, whose value is confirmed by the very low p-values (< 0.001 and < 0.01) in our analysis. Finally, the weight that the residual terms may have in SEM can be calculated and expressed by the fit index SRMR. In the present study the value of SRMR was only 0.078, which testifies that the weight of residual terms on the tested model is irrelevant and that SEM adequately confirms the theoretical path.

In view of the causal implications of climatic factors on the spread of SARS-CoV-2, it may be tempting to identify a threshold of solar irradiation or temperature or any other climatic factor, above which the COVID-19 spread is negatively affected. This thought is deeply appealing and was also one of our main goals before collecting, analyzing and, most importantly, understanding our data. In addition, several studies had already identified a cut-off value, although with very different results^2,8,10,14,29,33–38^.

However, in the case of COVID-19, considering only one climatic factor and trying to identify its threshold values, although potentially feasible, would be a constraint and, consequently, any resulting formula, would be conceptually wrong.

The correlation analyses of our study (reported in Table 2) and numerous meteorological studies incontrovertibly demonstrate that the main climatic factors (the 6 we considered) are interconnected. Therefore, focusing on single threshold values, above or below which a given event occurs, would have no mathematical or logical rationale, neither does (for example) trying to identify a threshold temperature value below which snowfall occurs. Snowfall does not depend only on temperature, but on the interaction of several atmospheric factors. Therefore, considering only the temperature will not lead to reliable forecasts since snow formation also depends on other factors (humidity, pressure, and wind). It is only through the calculation of all these variables together and the historical data collected over decades that the most likely scenarios can be identified.

Now, when it comes to SARS-CoV-2, the scenario becomes even more complex. In fact, not only is there a lack of reliable historical data on the epidemic in relation to climatic factors, but also there is the severely aggravating circumstance that, besides climatic factors, the spread of SARS-CoV-2 is influenced by countless other factors (population density, cultural habits, severity and observance of containment measures, intensity of trade and human contact, hygiene measures, etc.).

During our study, we soon realized that, due to the highly complex nature regulating the relationships between the variables, identifying a cut-off value was not feasible, precisely because too many causal variables are involved, each with a very wide range of variation. We thus decided to focus on the analysis of the relationships between climatic factors and virus spread, in order to identify the main players involved in the phenomenon and understand their effect. The magnitude and the algebraic sign of the factor loadings we calculated, led to understanding the type of correlation between each climatic variable and the latent variable Climate, identifying its strength and direction. In turn, the path coefficients linking Climate to the observed variable (incidence and prevalence) proved its correlation. Which is not negligible.

Basically, our study demonstrates that it is not possible to identify a mono-factorial threshold value to predict a reversal of COVID-19 spread. Only complex mathematical patterns (such as meteorological forecasting patterns), supported by an enormous amount of data sets (possibly also historical), may sufficiently define reliable predictability conditions.

In conclusion, the present study demonstrates that climatic factors significantly affect the spread of SARS-CoV-2 (probably and especially by influencing the air transmission through respiratory droplets). Among the variables investigated, solar irradiation proved to be the most influential factor, followed by temperature, humidity, precipitation, and, with a minimal or non-significant impact, pressure, and wind. However, demographic factors, together with other determinants, can affect the transmission (probably and especially through direct contact routes), and the influence of these can be such that they may overcome the protective effect of climate in some countries. Compared with the other studies reported in the literature, the present research has the merit of having addressed and overcome the limits of the previous investigations. To do so, however, it was necessary to use a robust mathematical model for the analysis (SEM), which required a considerable effort in terms of information needs, in order to function properly. The large amount of data used represents only a small part of the tremendous effort required, while greater effort was necessary for the selection of data and the evaluation of the criteria to be adopted for data processing. Only by respecting all these conditions, SEM could function properly and provide the results otherwise unattainable.

## Methods

### Data collection

Data regarding COVID-19 were collected from Johns Hopkins GitHub repository Systems Science and Engineering^23^. The information on governmental measures (school and university closures) were acquired from the UNESCO database^24^. The climatic parameters reported were taken from the dataset of the NASA Langley Research Center (LaRC) POWER Project^25^ and the demographic estimates (population size, land area, population density) were obtained from the United Nations population estimates and from the World Factbook of Central Intelligence Agency (CIA)^26^.

In order to evaluate the relation of COVID-19 with geo-climatic environment, the world has been divided into five geoclimatic zones, according to the updated Koppen-Geiger classification: polar, cold-temperate, warm- temperate, arid, and equatorial^27^.

For SARS-Cov-2 analysis, all the UN 193 countries have been taken into account. Among these, 16 small countries, with a population below one million and a density of less than 100 people/km^2^, as well as 18 countries with insufficient data on COVID-19 have been excluded. Conversely, all 50 states of the United States of America have been considered individually. Therefore, a total of 209 Countries have been included in the present study.

A total of 134,871 data were acquired from the sources mentioned above and inserted in a Microsoft Excel spreadsheet (Supplementary Table S2). Thirteen variables have been considered for the analysis and organized into the 3 groups herein reported.

1. Demographic: population size (number of people), land area (square kilometer—km^2^), population density (people/km^2^).
2. Climatic: climatic zone (one to five), temperature at two meters (degree Celsius—°C), solar irradiation (megajoule/square meter/day—MJ/m^2^/day), relative humidity (percentage—%), wind speed at two meters (meter per second—m/s), surface pressure (kilopascal—KPa), precipitation (millimeters/day—mm/day).
3. COVID-19: date of the first confirmed case, number of new weekly cases, and number of active weekly cases.

### Data processing

To ensure that the data collected met the purposes of the study, a set of specific criteria was established for the selection of the appropriate sample, and separate studies were performed to confirm the appropriateness of these choices. In particular:

1. Data on weekly new cases and active cases of SARS-CoV-2 infection were collected for a period of 16 weeks. Since the beginning of the infection did not occur simultaneously across all the countries, the data collected start from the first documented case in each country.
2. To evaluate the relationship between COVID-19 and climatic factors, matching epidemic and climatic data was found to be of importance. In each country, climatic conditions vary considerably across regions. Therefore, one to four cities, one for each of the regions most affected by COVID-19, were chosen for each country. Then, the weekly average was calculated for all the six climatic variables. Finally, the weekly means of all the cities were averaged to get the six total national weekly values. The process was repeated for all the weeks considered.
3. To evaluate the relationship between COVID-19 and climatic factors, a shift time between the collection of virologic data and the acquisition of climatic data had to be taken into consideration. In fact, the incubation period, the delay between symptom onset and testing, and the delay due to the communication of the result, contribute to a time shift between the infection exposure and the publication of the virologic data. Consequently, it is necessary to take into account a lag time between the collection of virologic data and the acquisition of climatic data for the analysis. According to the literature data^28–30^, a lag time of two weeks was considered in the present study.

### Data analysis

Data relative to SARS-CoV-2 were collected into a balanced panel dataset of 209 countries, starting from the first week of outbreak, until the sixteenth week. Due to data skewness (i.e. data with a non-Gaussian distribution), logarithmic transformation was applied to the analyzed variables.

The weekly incidence and prevalence of COVID-19 were calculated for each country, starting from the week of the first infection case and ending at week 16, according to the following formulas:

1 $${\text{Incidence}} = \frac{{{\text{Number }}\;{\text{of }}\;{\text{new}}\;{\text{ cases}}\;{\text{ of}}\;{\text{ Covid }}\;{\text{during }}\;{\text{week}}}}{{{\text{Population}}}} \times 100{,}000$$

2 $${\text{Prevalence}} = \frac{{{\text{Number}}\;{\text{ of }}\;{\text{confirmed }}\;{\text{cases }}\;{\text{per }}\;{\text{week}}}}{{{\text{Population}}}} \times 100{,}000$$

To verify whether the morbidity indices (incidence and prevalence) depend on the geoclimatic zones where countries are geographically located, the non-parametric k-sample test for median equality was applied and a geographic representation (thematic world map) was constructed for each of the explored variables.

To further investigate whether there is a correlation between incidence/prevalence and each climatic variable (considered individually), the Spearman rank correlation coefficient was calculated considering a two-week interval between the climatic data and the virological data. Furthermore, the correlation between incidence/prevalence and population density was also calculated.

Subsequently, on the basis of the results of the descriptive analysis, a path diagram was proposed (Fig. 2) to explore the potential relationships between meteorological variables, population density and morbidity indices. In this theoretical path it was assumed that meteorological factors were correlated to each other and linked to an unobserved/unmeasurable variable (or latent variable), indicated by the *Climate* label. Furthermore, it was hypothesized that *Climate* (that integrates the effects of meteorological variables) and *Population Density*, were regressors of *Incidence* and *Prevalence*, assuming the following pathways: *Climate* → *Incidence*, *Climate* → *Prevalence*, *Population Density* → *Incidence*, *Population Density* → *Prevalence*. Finally, *Incidence* and *Prevalence*, being closely connected by their nature, were linked by an arc that expresses their covariance.

To test this theoretical path and convert it into a set of equations, the authors applied the SEM, a broad and flexible statistical technique for modeling causal chain of effects simultaneously. Using a confirmatory approach (hypothesis-testing), this technique, examines the relationships between observed variables and not observed (latent) variables, in turn linked to observed variables, their indicators.

The SEM graphical representation is given by a path diagram, a kind of flow-chart that uses boxes, ellipses, and circles linked via arrows. Observed variables are represented by a box, and latent variables by an ellipse. Straight single-headed arrows express causal relations and double-headed curved arrows express correlations or covariance (without a causal interpretation). The values on the straight arrows between latent and observed variables and those between latent and observed indicators, represent, respectively, the path coefficients and the factors loading (the last being the correlation coefficient for the latent and observed indicators).

The circles with short arrows pointing to dependent variables refer to residual terms ε_i_. They include other possible variables that may influence dependent variables, but also random errors, errors in data entry, systematic errors leading to bias in the measurement of values. The value on the straight arrows between residual terms and dependent variables represents the residual variance (var ε_i_). Two important issues of SEM that deserve to be comprehensively addressed are the path coefficient and the residual variance. The path coefficient is a basic element in the SEM model. It indicates strength and direction of the causal impact of a variable considered as a cause on another variable that is considered, instead, an effect; it is like the regression coefficient. There are two types of path coefficients: non-standardized and standardized. Both express the influence of the causal variable on the dependent variable, but the first (the non-standardized one) reflects the change in terms of unit, while the second (the standardized), changes in terms of standard deviation. For example, in path A → B, a non-standardized path coefficient of 0.20 indicates that, if variable A increases by 1-unit, variable B is be expected to increase by 0.20 unit. In the case of standardized path coefficient, if variable A increases by one standard deviation from its mean, variable B is expected to increase by 0.20 its own standard deviation from its own mean. Frequently, SEM models include more than one variable as causative factor, often with different measurement units and order of magnitude. In these cases, the use of a non-standardized path coefficient is inappropriate because the effects of these variables on the dependent variable cannot be directly compared. Instead, the standardized path coefficient, being based on a normalised parameter (the standard deviation of the mean), allows for a direct comparison of the effects of variables, regardless of their original measurements.

Since both climatic variables and population density have very different scales of measurement and order of magnitude, in the present study the standardized path coefficient was considered for the analysis.

As for the residual variance, it is worth pointing out that it includes the effects of a lot of components (errors and other variables), each of which may have very little relevance. For its cumulative nature, residual variance may also reach high values, but the actual weight that it had in SEM is expressed by the fit index SRMR (reported below).

In order to reduce the random error, observations with missing values were excluded from the SEM model processing because the interpolation approach (often used for missing data) would have introduced other sources of errors in addition to those already present and due to (a) the large number of variables considered, (b) the large number of data processed, (c) the large number of Countries from which the data were collected, and (d) the heterogeneity of the same (often very different in a number of aspects).

To evaluate the adequacy of the model, the following fit indices were considered^31^: (a) coefficient of determination (CD) (similar to the R-squared value, ranging 0–1, good fit for values close to 1); (b) root mean square error of approximation (RMSEA) (good fit for RMSEA < 0.08), and (c) standardized root mean square residual (SRMR) (adequate fit for SRMR < 0.08).

SEM was fitted by maximum likelihood estimation (MLE) method and p-value less than 0.05 was considered as statistically significant. All of the statistical analysis was performed using STATA 14.0 (STATA Corp, College Station, TX)^32^, using the commands and the code reported below.

- Commands:
  *tsset command*, to structure in form of panel data the database containing the climate, socio-demographic and covid data of the 209 countries (for a total of 3764 records);
  *sem command*, to transform the relationships hypothesized between the variables (previously logarithmized) in a model of linear structural equations;
  *sem, standardize*, to obtain the standardized path coefficients;
- Code:
  *tsset id week_index*
  *sem *(*Climate* -> *L2.ln_Precipitation*,) (*Climate* -> *L2.ln_Humidity,*) (*Climate* -> *L2.ln_Pressure*,) (*Climate* -> *L2.ln_Temperature,*) (*Climate* -> *L2.ln_Wind*,) (*Climate* -> *L2.ln_Solar*,) (*Climate* -> *ln_Prevalence*,) (*Climate* -> *ln_Incidence,*) (*ln_Population_density* -> *ln_Incidence*,) (*ln_Population_density* -> *ln_Prevalence*,), *covstruct*(*_lexogenous, diagonal*) *cov*(_lexogenous*_oexogenous@0) *latent*(*Climate*) *cov*(e.ln_prevalence*e.ln_incidence)* nocapslatent*
  *sem, standardize*

## Supplementary Information

Supplementary Table S1.

Supplementary Table S2.

Supplementary TableS2 for STATA.
